# Gut microbial characteristical comparison reveals potential anti-aging function of *Dubosiella newyorkensis* in mice

**DOI:** 10.3389/fendo.2023.1133167

**Published:** 2023-01-31

**Authors:** Tian-hao Liu, Juan Wang, Chen-yang Zhang, Lin Zhao, Ying-yue Sheng, Guo-shui Tao, Yu-zheng Xue

**Affiliations:** ^1^ Affiliated Hospital of Jiangnan University, Wuxi, Jiangsu, China; ^2^ Medical College of Jiangnan University, Wuxi, Jiangsu, China; ^3^ Wuxi Traditional Chinese Medicine Hospital, Wuxi, Jiangsu, China

**Keywords:** aging, gut microbiota, *Dubosiella newyorkensis*, resveratrol, signal transduction

## Abstract

**Introduction:**

Previous study has indicated *Dubosiella newyorkensis* may act as a potential probiotic in age-related diseases. However, its detailed role in aging has not yet been promulgated. This study aimed to explore the potential anti-aging role of *Dubosiella newyorkensis* by comparing the anti-aging effect of resveratrol in young and old mice.

**Method:**

Measurement of intestinal aging-related factors in colon and serum, and vascular endothelial function-related factors in serum were performed by enzyme-linked immunosorbent assay (ELISA). Gut microbial analysis of intestinal contents were identified by 16S rRNA gene sequencing.

**Results:**

The effect of *Dubosiella newyorkensis* on reducing malondialdehyde (MDA) and increasing superoxide dismutase (SOD) in aged mice were greater than that of resveratrol. While the effect of *Dubosiella newyorkensis* on nitric oxide (NO) level was less than that of resveratrol, the reduction of vascular endothelial growth factor (VEGF) and pentosidine (PTD) was better than that of resveratrol in young mice. In young mice, *Dubosiella newyorkensis* promoted an increase in the beneficial genus *Lactobacillus, Bifidobacterium* and *Ileibacterium* less effectively as compared with resveratrol treatment. In aged mice, *Dubosiella newyorkensis* promoted the increase of *Bifidobacterium, Ileibacterium* less effectively than resveratrol, and promoted the increase of *Akkermansia, Staphylococcus, Verrucomicrobiota* expression better as compared with resveratrol treatment. Both young and old mice showed the same results for the remaining markers, including changes in gut microbial composition and predictions of function.

**Conclusion:**

*Dubosiella newyorkensis* has similar anti-aging functions with resveratrol. *Dubosiella newyorkensis* may even be more effective than resveratrol in reducing oxidative stress, improving vascular endothelial function, and redistributing gut microbiota. The research provides an innovative strategy of *Dubosiella newyorkensis* to improve aging.

## 1 Introduction

Aging is a gradual and unavoidable step in the metabolic process of the organism, characterized by a degenerative change in the organism’s degree of health and capacity to maintain its own internal homeostasis, indicated by a loss of the organism’s ability to adapt to its environment. It is a multi-linked biological process that results from the combined action of several causes, and its processes are highly complicated, including changes in the structure and function of various organ systems. These alterations can raise the likelihood of biological death and are frequently followed with illness development (1). As the population ages, the incidence of diseases associated with aging will gradually increase (2, 3) and cardiovascular disease is the current leading worldwide cause of mortality (4). Aging is inevitable, so the search for effective anti-aging techniques and methods to delay the destruction of aging, significantly improve the quality of life of the elderly, and reduce the burden on families, society, and the nation is a top priority for society and scientists. There are more than 300 theories about aging (5), but most of them are not credible, among which the accepted theory of oxidative stress suggests that biological macromolecules damage, including DNA, proteins and lipid peroxidation is a predisposing event for aging (6–8). Oxidative stress can also lead to endothelial nitric oxide synthase (eNOS) dysregulation and vascular endothelial dysfunction, thereby inducing vascular senescence (9). Oxidative stress-related endothelial damage is the initiating factor of cardiovascular disease (10, 11). The gut microbial community and the human body are in a mutually beneficial symbiotic relationship; however, aging alters the composition of the gut microbiota. Compared to healthy young adults, the gut microbial diversity generally decreases in older adults, and the number of pathogenic gut microbiota increases significantly. Alterations in the microbiota lead to damage to the intestinal barrier and increased intestinal permeability, induce the production of inflammatory substances, stimulate the host systemic immune response, and ultimately result in a long-term chronic pro-inflammatory state in the organism, damaging the immune system and leading to mutated and senescent cells that cannot be cleared properly (12). Vascular health and gut microbiota play a key role in human aging. *Dubosiella* is a potential probiotic found in our preliminary research to improve obesity, hypertension and liver disease (13). *Dubosiella newyorkensis* is a stain of *Dubosiella* identified in 2017 (14). The abundances of *Dubosiella newyorkensis* were greatly reduced in APP^swe^/PS1^ΔE9^ (PAP) mice with cognitive decline and age (15). But the evidence of correlation between *Dubosiella* with aging is not strong.

Resveratrol is recognized as an anti-aging wonder drug, and there have been numerous studies showing that it can improve oxidative stress and vascular endothelial function and regulate intestinal microbiota thus achieving anti-aging effects (16–19). Therefore, this study was conducted to investigate the anti-aging potential of *Dubosiella newyorkensis* by comparing the intervention of resveratrol and *Dubosiella newyorkensis*, in two animal models of young and aged mice. This study revealed that *Dubosiella newyorkensis* and resveratrol have similar anti-aging functions. Even, *Dubosiella newyorkensis* may be superior to resveratrol in reducing oxidative stress, improving vascular endothelial function and improving gut microbiota distribution. The research provides an innovative strategy to improve aging.

## 2 Methods

### 2.1 Design and grouping

A total of sixteenth C56BL/6J mice (6-8 weeks old, male, provided by Changzhou cavens experimental animal Co., Ltd.) were randomly divided into resveratrol intervention young group (Resveratrol_young) and *Dubosiella newyorkensis* intervention young group (TSD_64_young). Meanwhile, sixteenth 14-month-old C56BL/6J mice (male, provided by Changzhou cavens experimental animal Co., Ltd.) were randomly designed as older of resveratrol intervention (Resveratrol_aged) and older of *Dubosiella newyorkensis* intervention (TSD_64_aged), with 6 mice in each group for experimental validation. The ethics committee of Jiangnan University approved the animal experiment (NO.20211015c0650220).

### 2.2 Intervention

A normal diet and free drink water for 4 weeks were performed in the all groups. Nantong Troffer Feed Technology Co., Ltd. (Nantong, China) provided the feed (production license (2014): 06092), which was then sterilized by Nantong Michael Irradiation Co., Ltd. (Nantong, China). Resveratrol intervention in young and aged mice were gavaged using resveratrol (44 mg/kg/day), while the mice in young and aged groups for *Dubosiella newyorkensis* intervention were administered by gavage with the same 100 μL *Dubosiella newyorkensis*. *Dubosiella newyorkensis* was NYU-BL-A4 (ATCC: TSD_64) provided by the International Conservation Center, qualified by Guangdong Institute of Microbiology, and cultured in 10^8^ cfu with Guangdong Institute of Microbiology.

### 2.3 Measurement of intestinal aging-related factors in colon and serum by enzyme-linked immunosorbent assay (ELISA)

To reduce suffering, isoflurane anesthesia was administered to all mice after 4 weeks. After that, blood was extracted from the eyeball and centrifuged after 2-4 h for 5 min at 3000 rpm. The supernatant was then sub packed for storage into 1.5-mL sterilized EP tubes. The colon tissue (1-2 cm) was gathered and stored in the EP tubes. Lastly, the levels of malondialdehyde (MDA), catalase (CAT), glutathione peroxidase (GSH-Px), and superoxide dismutase (SOD) in the colon and serum were assessed in accordance with the kit’s instructions, which were bought from Jiangsu Meimian Industrial Co., Ltd (Yancheng, China).

### 2.4 Detection of vascular endothelial function-related factors in serum by ELISA

Then, using kits provided by Jiangsu Meimian Industrial Co., Ltd (Yancheng, China) and following the operating instructions, the levels of nitric oxide (NO), endothelin-1 (ET-1), angiotensin II (AngII), vascular endothelial growth factor (VEGF), vascular cell adhesion molecule-1 (VCAM-1) and pentosidine (PTD) were determined.

### 2.5 16S rRNA gene sequencing, gut microbial analysis of intestinal contents

The intestinal contents of 6 mice in each group were randomly selected for subsequent analysis. The intestinal contents were used to extract microbial DNA through E.Z.N.A.^®^ soil DNA Kit (Omega Bio-Tek, Norcross, GA, U.S.). The final DNA concentration and purification was assessed using NanoDrop 2000 UV-vis spectrophotometer (Thermo Scientific, Wilmington, USA), and DNA quality was estimated using 1% agarose gel electrophoresis. The V3-V4 hypervariable portions of the bacterium 16S rRNA gene were amplified by a thermocycler PCR system (GeneAmp 9700, ABI, USA) with primers 338F (5’-ACTCCTACGGGAGGCAGCAG-3’) and 806R (5’-GGACTACHVGGGTWTCTAAT-3’). The PCR products were extracted from a 2% agarose gel, purified with a AxyPrep DNA Gel Extraction Kit (Axygen Biosciences, Union City, CA, USA), and quantified with QuantiFluorTM-ST (Promega, USA) according to the manufacturer’s instructions.

The purified amplicons were sequenced on an Illumina MiSeq platform (Illumina, San Diego, USA) at an equimolar ratio (Shanghai, China). The operational taxonomic units (OTUs) were clustered using UPARSE (http://drive5.com/uparse/) with a unique ‘greedy’ technique that performs chimera filtering and OTU clustering at the same time. Finally, the RDP Classifier algorithm was used to compare the taxonomy of each 16S rRNA gene sequence to the 16S rRNA database (Silva (SSU123)).

### 2.6 Statistical analysis

GraphPad Prism software was used to analyze all data, which were all expressed as mean standard error (SEM). The Kruskal-Wallis test, one-way ANOVA, unpaired T-test were used for all analyzes. *P* < 0.05 means a meaningful difference. The Majorbio I-Sanger Cloud Platform (www.i-sanger.com) was utilized to examine the 16S rRNA gene sequencing data.

**Figure 1:**
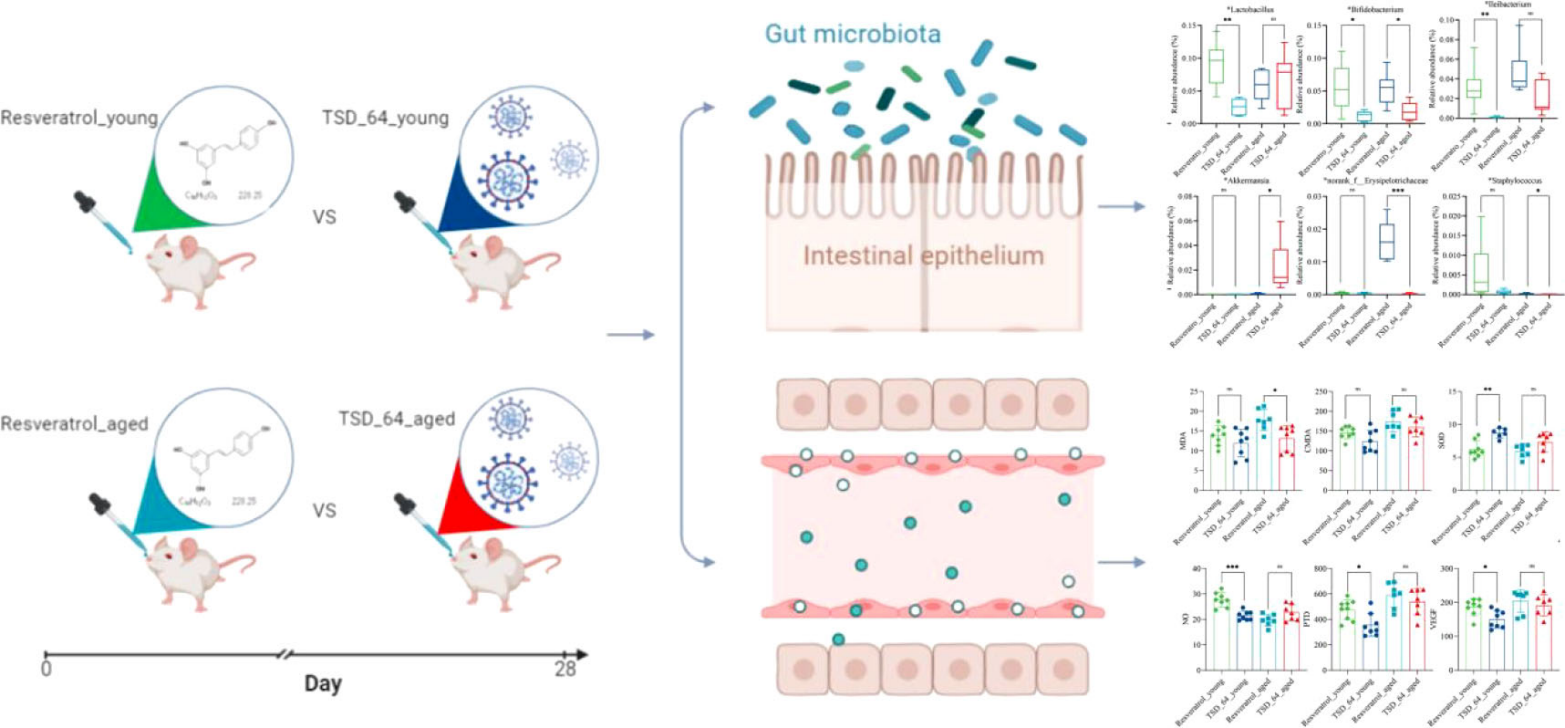
Flow chart of the study. Young and aged mice were gavaged using resveratrol and *dubosiella newyorkensis*. Colon and serum samples were collected for testing indexes of oxidative stress, vascular endothelial and gut microbiota, to analyze the similarities and differences of resveratrol and *dubosiella newyorkensis*. The group of young mice gavaged with resveratrol was named Resveratrol_young; The group of young mice gavaged with *dubosiella newyorkensis* was named TSD_64_young; The group of aged mice gavaged with resveratrol was named Resveratrol_aged; The group of young mice gavaged with *dubosiella newyorkensis* was named Resveratrol_aged.

A brief operation flow was shown in **Figure 1**.

## 3 Results

### 3.1 Changes in oxidative stress in mice

**Figure 2:**
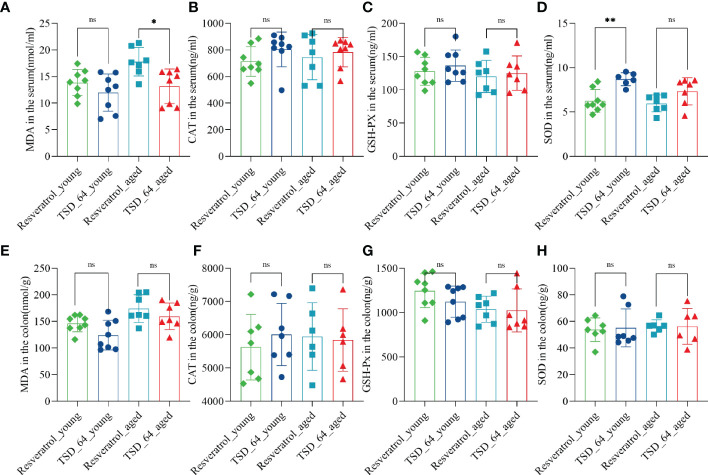
Changes in oxidative stress in the colon and serum in mice. **(A)** The level of MDA in serum. **(B)** The level of CAT in serum. **(C)** The level of GSH-PX in serum. **(D)** The level of SOD in serum. **(E)** The level of MDA in colon. **(F)** The level of CAT in colon. **(G)** The level of GSH-PX in colon. **(H)** The level of SOD in colon. Malondialdehyde (MDA), catalase (CAT), glutathione peroxidase (GSH-Px), and superoxide dismutase (SOD) in the colon and serum were assessed. The values are expressed as mean ± standard deviation. “ns” represented no significant difference, *P<0.05, **P<0.01. The group of young mice gavaged with resveratrol was named Resveratrol_young; The group of young mice gavaged with *dubosiella newyorkensis* was named TSD_64_young; The group of aged mice gavaged with resveratrol was named Resveratrol_aged; The group of young mice gavaged with *dubosiella newyorkensis* was named Resveratrol_aged. N=6-8 per group.

As the results shown, in the aged mice experiment, the reduction of MDA in serum was significantly more pronounced in TSD_64_aged group than Resveratrol_aged group (**Figure 2A**), and in young mice experiment, the effect of elevated SOD in serum was better in TSD_64_young group than Resveratrol_young group, and the difference was statistically significant (**Figure 2D**). No difference in the effect of changing the level of CAT and GSH-Px in the serum (**Figures 2B, C**). and also no difference in the effect of changing the level of those indicators in the colon (**Figures 2E–H**). It indicates that *Dubosiella newyorkensis* may have similar functions to resveratrol in improving oxidative stress and is superior with resveratrol in reducing the oxidative stress indicator MDA and increasing antioxidant enzyme SOD in the serum, and no difference in regulation for intestinal stress.

### 3.2 Changes of vascular endothelial function in mice

**Figure 3:**
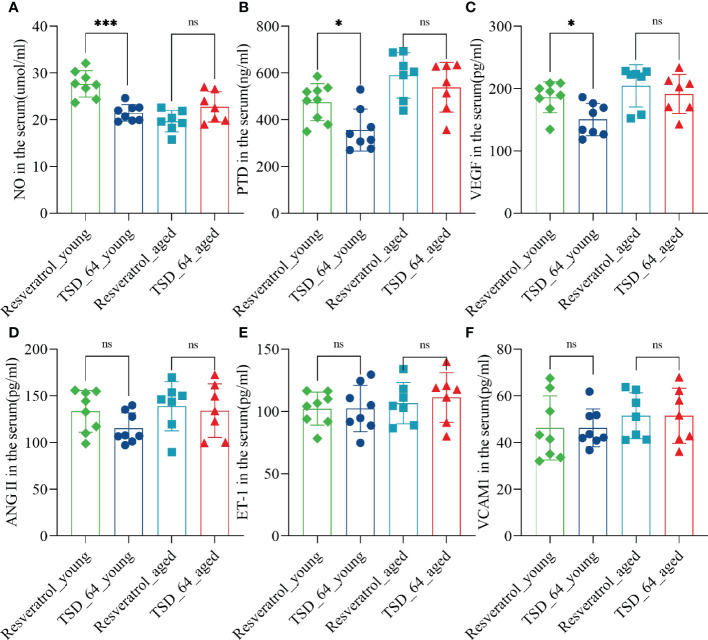
Changes of vascular endothelial function in mice. **(A)** The levels of NO. **(B)** The levels of PTD. **(C)** The levels of VEGF. **(D)** The levels of ANG II. **(E)** The levels of ET-1. **(F)** The levels of VCAM1. nitric oxide (NO), endothelin-1 (ET-1), angiotensin II (Ang II), vascular endothelial growth factor (VEGF), vascular cell adhesion molecule-1 (VCAM-1) and Pentosidine (PTD) were assessed. The values are expressed as mean ± standard deviation. “ns” represented no significant difference, *P<0.05, ***P<0.001. The group of young mice gavaged with resveratrol was named Resveratrol_young; The group of young mice gavaged with *dubosiella newyorkensis* was named TSD_64_young; The group of aged mice gavaged with resveratrol was named Resveratrol_aged; The group of young mice gavaged with *dubosiella newyorkensis* was named Resveratrol_aged. N=6-8 per group.

The results of the endothelial functional status index showed that NO levels of increasing in the TSD_64_young group was not as great as that in Resveratrol_young group (**Figure 3A**), but in reducing two indicators, VEGF and PTD, TSD_64_young group was better than Resveratrol_young group (**Figures 3B, C**). There was no difference in the rest of the indicators (**Figures 3D–F**). It is thus clear that *Dubosiella newyorkensis* also had similar effects to resveratrol in improving vascular function. Moreover, *Dubosiella newyorkensis* has less vasodilatory capacity than resveratrol, but is stronger than resveratrol in inhibiting the proliferation of vascular endothelial cells and in resisting the synthesis of advanced glycosylation end products (AGEs) pentosidine. In summary, *Dubosiella newyorkensis* has similar effects to resveratrol in improving oxidative stress and vascular endothelial function. Therefore, it was further investigated whether its changes of gut microbiota has similarities.

### 3.3 Overall structure and composition of gut microbiota

#### 3.3.1 Changes in OTU count and diversity of gut microbiota in mice

**Figure 4:**
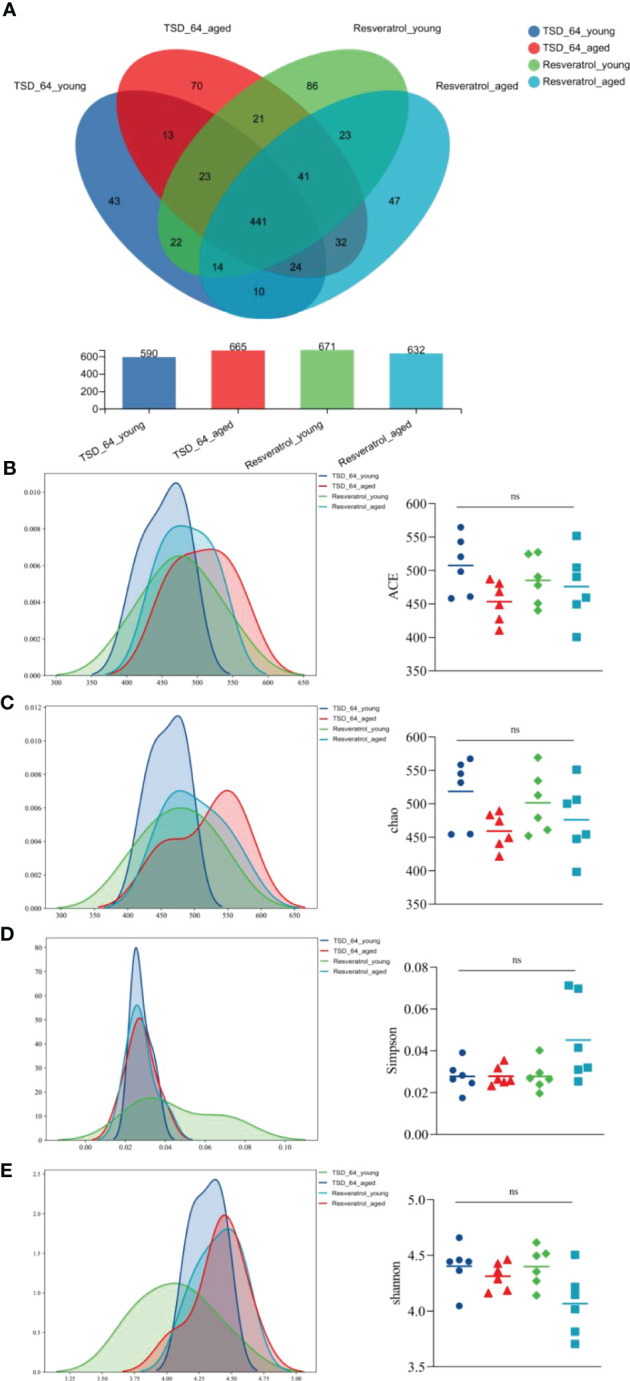
Changes in OTU count and alpha diversity of gut microbiota in mice. **(A)** Venn diagram of the OTU counts in the four groups. **(B)** Abundance-based Coverage Estimator (ACE). **(C)** Chao1. **(D)** Simpson index (Simpson). **(E)** Shannon-Wiener index(Shannon). The abundance index (community richness) was assessed using ACE and Chao to reflect the abundance of species within the community. A larger ACE index indicates a larger number of species in the community. The greater the chao index, the greater the number of OTUs, indicating a higher number of species in a community. The species diversity index was assessed using simpson and shannon reflects the combined status of species richness and evenness. Higher Shannon index values indicate higher alpha diversity in the community. Larger Simpson index values indicate lower community diversity. The values are expressed as mean ± standard deviation. “ns” represented no significant difference. The group of young mice gavaged with resveratrol was named Resveratrol_young; The group of young mice gavaged with *dubosiella newyorkensis* was named TSD_64_young; The group of aged mice gavaged with resveratrol was named Resveratrol_aged; The group of young mice gavaged with *dubosiella newyorkensis* was named Resveratrol_aged. N=6 per group.

The Venn diagram analyzed the unique or common operational taxonomic units(OTUs) between the different sample groups, visualizing the similarity and uniqueness at the OTU level. Eighty OTUs were common to the four groups. Eighty-six OTUs were unique to the Resveratrol_young group, with a total of 671 OTUs, and forty-three were unique to the TSD_64_young group, with a total of 665 OTUs. This suggests that in the young group, *Dubosiella newyorkensis* reduced the number of gut microbial species compared to resveratrol. Forty-seven OTUs were unique to the Resveratrol_aged group, with a total of 632 OTUs. Seventy were unique to the TSD_64_aged group, with a total of 632 OTUs (**Figure 4A**). This suggests that in the aged group, *Dubosiella newyorkensis* increased the number of gut microbial species compared to resveratrol. Species richness was determined by ACE and Chao. Shannon and Simpson indices were used to assess the diversity of the community, with an overall assessment of α-diversity (**Figures 4B–E**). ACE, Chao and Shannon indices were slightly higher in the TSD_64_young group than in the Resveratrol_young group (*p* > 0.05, *p* > 0.05, and *p* > 0.05). The Simpson index of the TSD_64_young group has no difference from that of the Resveratrol_young group (*p* > 0.05). ACE, Chao, and Simpson indices were slightly lower in the TSD_64_aged group than in the Resveratrol_aged group (*p* > 0.05, *p* > 0.05, and *p* > 0.05). The Shannon index of the TSD_64_aged group has no difference from that of the Resveratrol_aged group (*p* > 0.05).

**Figure 5:**
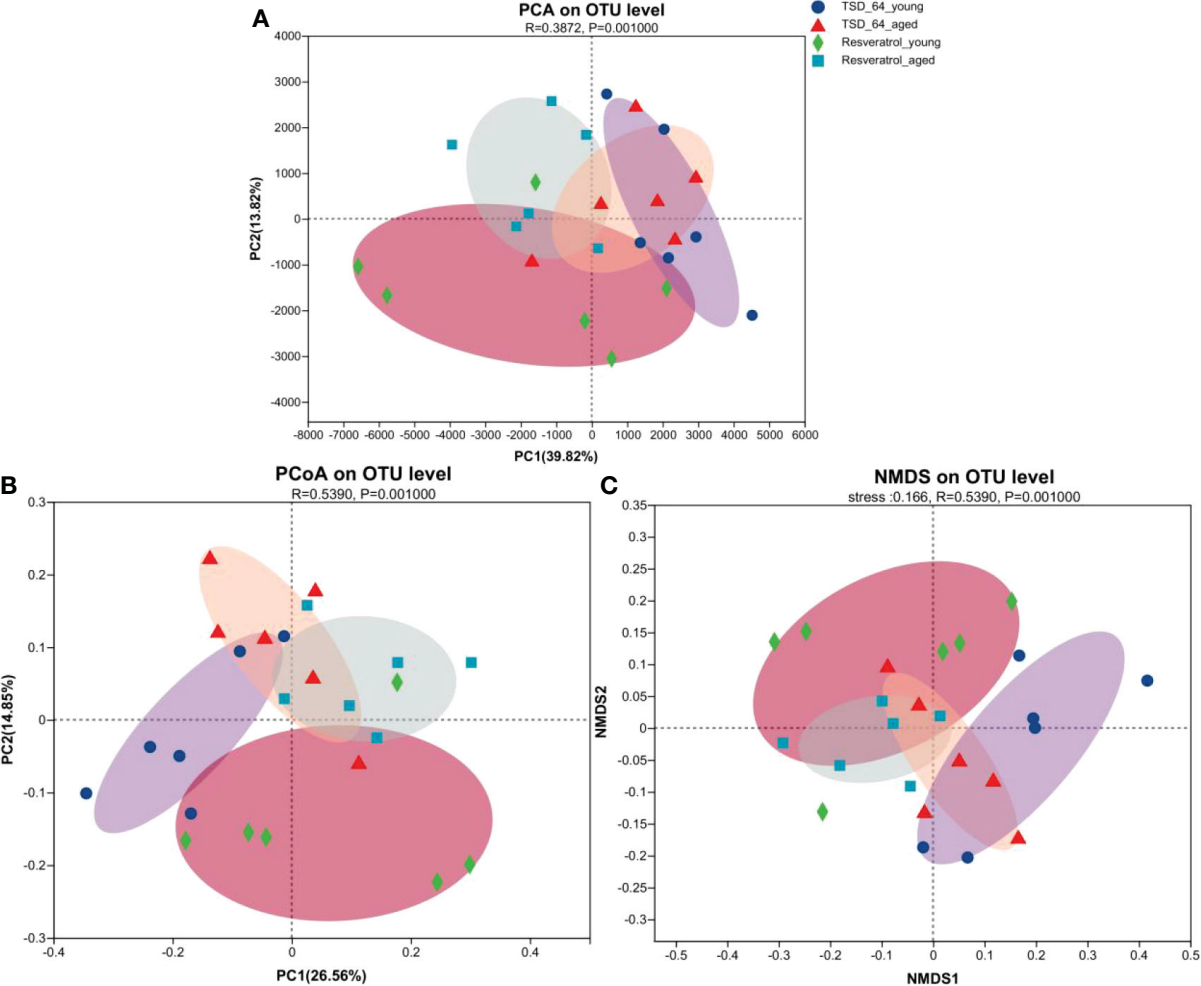
Changes in beta diversity of gut microbiota in mice. **(A)** Principal Components Analysis (PCA). **(B)** Principal Co-ordinates Analysis (PCoA). **(C)** Non-metric multidimensional scaling (NMDS). Unconstrained PCoA (for principal coordinates PCo1 and PCo2) and Unconstrained PCA (for principal coordinates PC1 and PC2) with Bray-Curtis distance showing that the gut microbiota of Resveratrol_young and Resveratrol_aged separate from those of TSD_64_young and TSD_64_young in the first axis. NMDS plots are presented based on Bray-Curtis similarity. The 2D stress value for each panel ranges between 0.11-0.18. TSD_64_young communities are presented with round symbols, TSD_64_aged communities with triangle symbols, Resveratrol_young communities with rhombus symbols and Resveratrol_aged communities with square symbols. In each panel, smaller symbols depict individual samples. *P*=0.001, permutational multivariate analysis of variance (PERMANOVA) by Adonis. The group of young mice gavaged with resveratrol was named Resveratrol_young; The group of young mice gavaged with *dubosiella newyorkensis* was named TSD_64_young; The group of aged mice gavaged with resveratrol was named Resveratrol_aged; The group of young mice gavaged with *dubosiella newyorkensis* was named Resveratrol_aged. N=6 per group.

Alpha-diversity analysis showed that no significant differences in microbial community were observed between the four groups. β-diversity analysis by PCoA, PCA, NMDS (**Figures 5A–C**) was used to further understand the effect of *Dubosiella newyorkensis* and resveratrol on the overall structural changes of the intestinal microbial community. Samples from the TSD_64_young group were effectively separated from those from the Resveratrol_aged group, showing a clear grouping phenomenon. However, samples from the TSD_64_aged group showed grouping and aggregation with samples from the Resveratrol_aged group. All these findings suggest that the use of both resveratrol and *Dubosiella newyorkensis* significantly modulates the intestinal microbial structure of mice. The small difference between resveratrol and *Dubosiella newyorkensis* in aging mice better illustrates the similarity between the two in terms of anti-aging.

#### 3.3.2 Changes in the composition of the gut microbiota

**Figure 6:**
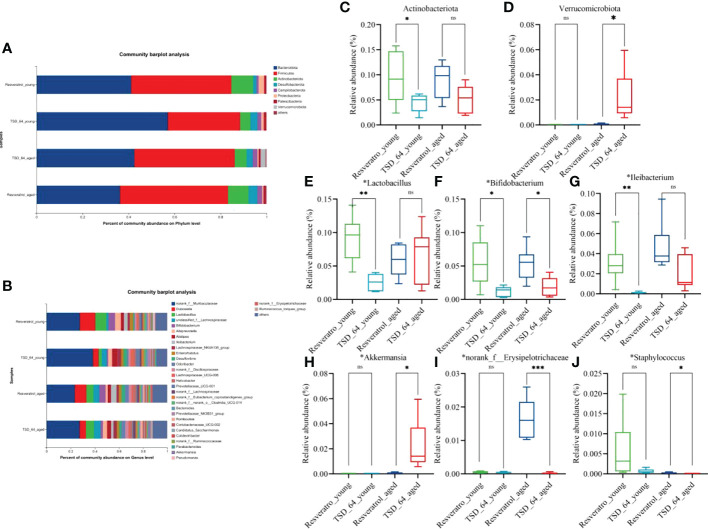
Changes in the composition of the gut microbiota. **(A)** Horizontal bar diagram of the phylum level. **(B)** Horizontal bar diagram of the genus level. **(C)** Relative composition abundance of *Actinobacteria*. **(D)** Relative composition abundance of *Verrucomicrobiota*. **(E)** Relative composition abundance of *Lactobacillus*. **(F)** Relative composition abundance of *Bifidobacterium*. **(G)** Relative composition abundance of *Ileibacterium*. **(H)** Relative composition abundance of *Akkermansi.***(I)** Relative composition abundance of *norank_f:Muribaculaceae*. **(J)** Relative composition abundance of *Staphylococcus.* The values are expressed as mean ± standard deviation. Statistical tests using unpaired t-tests. “ns” represented no significant difference, *P<0.05, **P<0.01, *** *P<*0.001. The group of young mice gavaged with resveratrol was named Resveratrol_young; The group of young mice gavaged with *dubosiella newyorkensis* was named TSD_64_young; The group of aged mice gavaged with resveratrol was named Resveratrol_aged; The group of young mice gavaged with *dubosiella newyorkensis* was named Resveratrol_aged. N=6 per group.

To further investigate the specific changes in gut microbial composition induced by the two interventions in the young and aged mice, we analyzed the differences in taxonomic composition at both the phylum and genus levels. As shown, at the phylum level, the intestinal microbiota of all experimental groups consisted mainly of *Firmicutes* and *Bacteroidetes* (**Figure 6A**). The relative abundance of *Actinobacteria* was significantly lower in the TSD_64_young group compared with the Resveratrol_young group (**Figure 6C**), and in aged mice, the relative abundance of *Verrucomicrobiota* was significantly increased in the TSD_64_aged group compared to the Resveratrol_aged group (**Figure 6D**). At the genus level, the groups were mainly composed of *norank_f:Muribaculaceae, Dubosiella*, followed by *Lactobacillus, unclassified_f:Lachnospiraceae* (**Figure 6B**). At the genus level, A significant decrease in the relative abundance of *Lactobacillus, Bifidobacterium* and *Ileibacterium* could be observed in the TSD_64_young group compared to the Resveratrol_young intervention group. the relative abundance of *Bifidobacterium, norank_f:Erysipelotrichaceae* and *Staphylococcus* decreased in the TSD_64_aged group compared to the Resveratrol_aged group, while the relative abundance of *Akkermansiaceae* increased (**Figures 6E–J**). The differences in the abundance of the other phylum and genus levels were not significant. This suggests that there are similarities in the composition of the gut microbiota affected by *Dubosiella newyorkensis* and resveratrol, with *Dubosiella newyorkensis* being less effective than resveratrol in increasing the relative abundance of beneficial bacteria, but more effective in reducing the relative abundance of harmful bacteria.

#### 3.3.3 Analysis of significantly different gut microbiota

**Figure 7:**
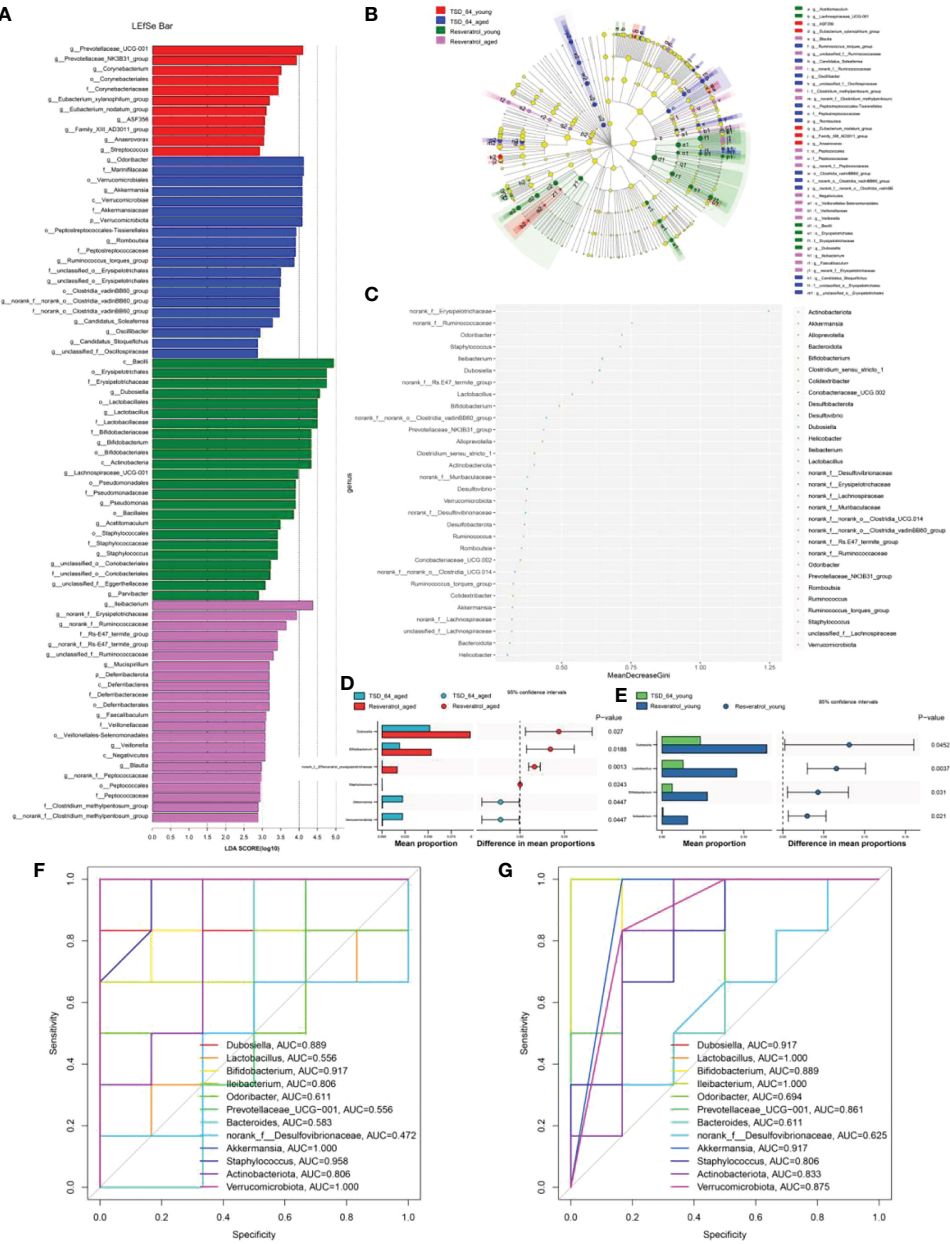
Analysis of significantly different gut microbiota in mice. **(A)** LDA Effect Size (LEfSe) analysis. **(B)** Cladogram. **(C)** Random Forest. **(D)** STAMP analysis in young mice. **(E)** STAMP analysis in aged mice. **(F)** ROC curve in young mice. **(G)** ROC curve in aged mice. The LEfSe analysis mainly shows us the significantly different species with LDA scores greater than the preset value, i.e., statistically different Biomaker, with a default preset value of 2.0. Cladogram shows us each small circle at a different taxonomic level represents a taxon at that level, and the diameter of the small circles represents the relative abundance. Species with no significant differences are uniformly colored yellow, species with significant differences Biomarker follows the group coloring, for example, red nodes indicate microbial taxa that play an important role in the red group, and so on. Species names corresponding to Biomarkers not shown in the figure are displayed on the right side, with letter numbers corresponding to those in the figure. Random forest for feature importance ranking and feature selection. STAMP analysis: the bar chart on the left shows the difference in values between the two groups. The dotted bar graph on the right side shows the percentage of species between the two groups for all species in the two sample groups, respectively. As long as the area under the ROC curve is greater than 0.7, it proves that the diagnostic test has some diagnostic value. The group of young mice gavaged with resveratrol was named Resveratrol_young; The group of young mice gavaged with *dubosiella newyorkensis* was named TSD_64_young; The group of aged mice gavaged with resveratrol was named Resveratrol_aged; The group of young mice gavaged with *dubosiella newyorkensis* was named Resveratrol_aged. N=6 per group.

Further, we analyzed the similarities and differences of significantly different microbiota among different groups and different interventions. The results of LDA Effect Size analysis showed that the expression of *Prevotellaceae_UCG-001* was significant in TSD_64_young group, and *Bacilli, Erysipelotrichales, Erysipelotrichaceae, Dubosiella, Lactobacillales, Lactobacillus, Lactobacillaceae, Bifidobacteriaceae, Bifidobacterium, Bifidobacteriales, Actinobacteria* were significantly expressed in Resveratrol_young group. In aged mice, *Odoribacter, Marinifilaceae, Verrucomicrobiales, Akkermansia, Verrucomicrobiae, Akkermansiaceae, Verrucomicrobiota* were significantly expressed in the TSD_64_aged group, and *Ileibacterium* was significantly expressed in the Resveratrol_aged group (**Figures 7A, B**). Random forest plots showing the order of importance (**Figure 7C**) were taken to intersect with the significantly expressed groups in the LEFSe analysis to obtain *Odoribacter, Ileibacterium, Dubosiella, Lactobacillus, Bifidobacterium, Verrucomicrobiota, Akkermansia. Akkermansia*. Further Stamp analysis revealed that in young mice, *Dubosiella, Lactobacillus, Bifidobacterium* and *Ileibacterium* were relatively low expressed in the TSD_64_young group compared to the Resveratrol _young group (**Figure 7E**). *Dubosiella, Bifidobacterium, norank_f:EResveratrol_youngsipelotrichacea* were relatively low expressed in the TSD_64_aged group compared to the Resveratrol_aged group, whereas *Staphylococcus, Akkermansia* and *Verrucomicrobiota* were relatively highly expressed (**Figure 7D**). The results were shown by ROC curves (**Figures 7F, G**). Compared with the Resveratrol_young group, *Dubosiella, Lactobacillus, Bifidobacterium, Ileibacterium, Prevotellaceae_UCG-001, Akkermansia, Staphylococcus, Actinobacteriota, Verrucomicrobiota*, AUC > 0.7, especially the significantly expressed genus *Lactobacillus* and *Prevotellaceae_UCG-001*, which is unique to young mice, could serve as a biological annotator for the intervention of *Dubosiella newyorkensis*, but its expression did not differ between the two drug interventions. *Dubosiella, Bifidobacterium, Ileibacterium, Akkermansia, Staphylococcus, Actinobacteriota, Verrucomicrobiota*, AUC > 0.7 in the TSD_64_aged group compared to the Resveratrol_aged group. These gut microbiota served as microbial markers for the *Dubosiella newyorkensis* intervention in aged mice. In conclusion, in young mice, *Dubosiella newyorkensis* promoted an increase in the beneficial genus *Lactobacillus, Bifidobacterium* and *Ileibacterium* less effectively than resveratrol. In aged mice, *Dubosiella newyorkensis* promoted the increase of *Bifidobacterium, Ileibacterium* less effectively than resveratrol, and promoted the increase of *Akkermansia, Staphylococcus, Verrucomicrobiota* expression better than resveratrol. Therefore, *Dubosiella newyorkensis* was similar to the effect of resveratrol on gut microbiota, can remodel the gut microbiota as well as resveratrol. Thus, it can promote the abundance of thick-walled bacterial phylum and increase the beneficial bacteria and thus act.

### 3.4 Predict function

**Figure 8:**
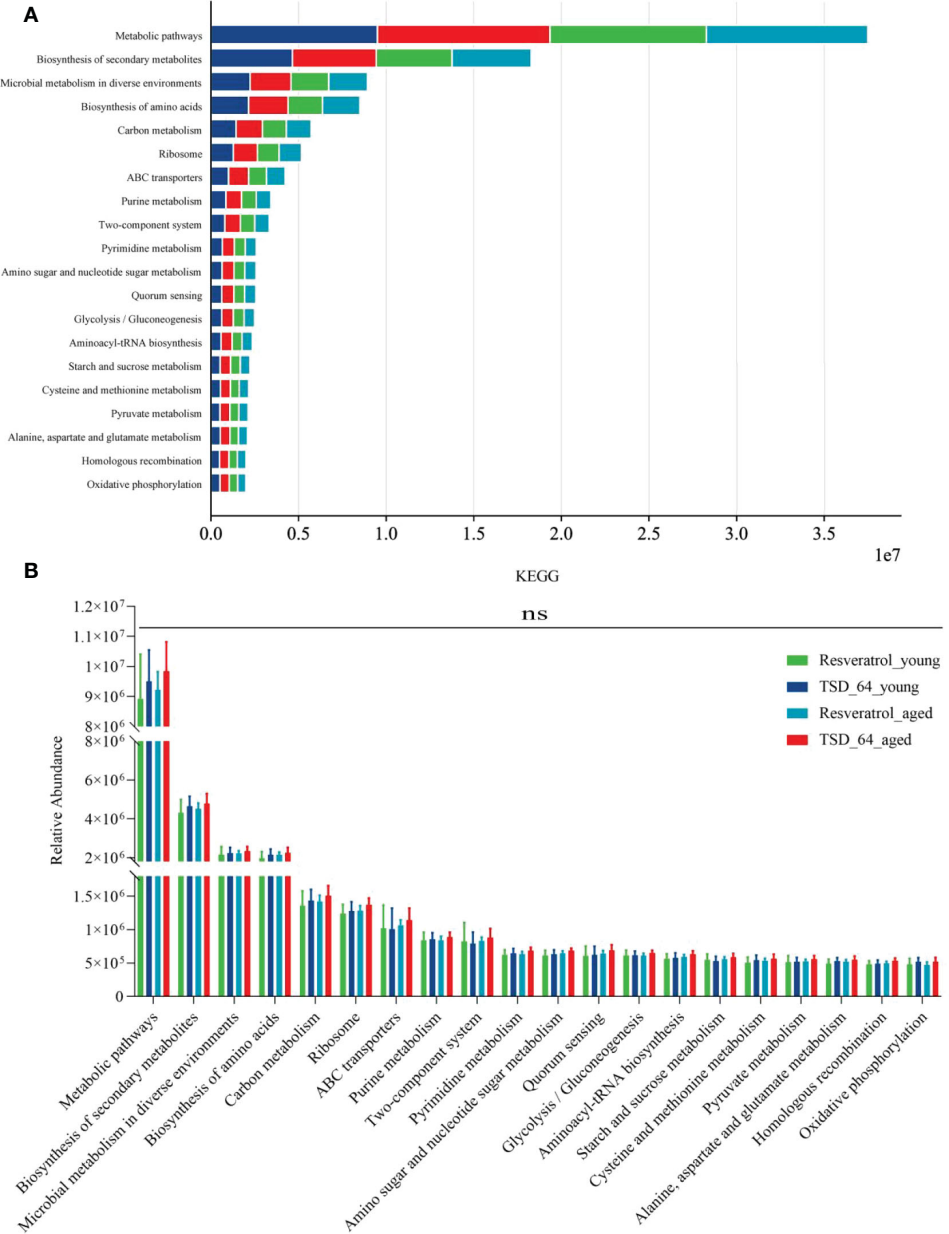
Prediction of function. **(A)** Visualization the top 20 KEGG pathways between the four groups of composition. **(B)** Unpaired t-test for the top 20 KEGG pathways between the four groups of composition. The values are expressed as mean ± standard deviation. Statistical tests using unpaired t-tests. “ns” represented no significant difference, *P<0.05, **P<0.01, ***P<0.001. The group of young mice gavaged with resveratrol was named Resveratrol_young; The group of young mice gavaged with *dubosiella newyorkensis* was named TSD_64_young; The group of aged mice gavaged with resveratrol was named Resveratrol_aged; The group of young mice gavaged with *dubosiella newyorkensis* was named Resveratrol_aged. N=6 per group.

The results of function prediction showed that a total of 356 KEGG pathways were enriched, visualizing the top 20 pathways (**Figure 8A**), and an unpaired t-test between groups for the top 20 significantly enriched KEGG pathways revealed no difference in enrichment between the two groups of mice for the two interventions (**Figure 8B**). This suggests that may have the same biological role as resveratrol and be involved in the same regulatory pathways. It may be because *Dubosiella newyorkensis* and the positive drug resveratrol have similar pathways of action so there is no difference in functional pathways. In conclusion, *Dubosiella newyorkensis* may exert the same biological functions as resveratrol.

## 4 Discussion

Some of the more successful pre-anti-aging attempts include calorie restriction (especially the mTORC1 signaling pathway), removal of senescent cells, reversal of stem cell senescence, fecal transplantation (microbiome suppression), guided autophagy, and reduction of inflammation (20). The most mainstream approach is to eliminate senescent cells with the latest discoveries of molecular compounds that remove senescent cells, including metformin, resveratrol, spermidine, rapamycin, NAD + supplements, and senolytics (21). Among them, resveratrol comes from grape skin, grape seed, peanut coat and thuja extract. It is a plant-derived polyphenol with antioxidant, free radical scavenging and anti-aging effects, and is recognized as an anti-aging wonder drug (22, 23). Study shows resveratrol can effectively scavenge ROS accumulation (24, 25) and increases antioxidant defenses (26, 27). Evidence from studies shown that an increase or restoration in the level of CAT, GPx, GR, and GSH was more pronounced when resveratrol was administered in HCC (28). Another study also showed that restoration could enhance SOD, GSH-Px, and CAT activities and HO-1 protein levels and decrease MDA content were detected in the brain tissue of the Res-treated mice (29). Study shows that resveratrol significantly reduces MDA levels (30). So here, the activities of the antioxidant enzymes GPx, SOD and CAT and the levels of the small molecule MDA were used to assess the levels of oxidative stress in tissues and serum. The results showed that *Dubosiella newyorkensis* was superior to resveratrol in lowering the index MDA in aging mice, while elevating the antioxidant enzyme SOD in young mice was superior to the oxidative stress index. This indicates that *Dubosiella newyorkensis* has superior antioxidant potential to resveratrol. *In vitro* cell culture studies revealed that resveratrol promotes eNOS activity, which helps catalyze the production of NO by vascular endothelial cells in order to play a role in maintaining arterial diastole (31). Studies with endothelial cells cultured *in vitro* revealed that resveratrol inhibits the expression of adhesion molecules in endothelial cells (32). Resveratrol reverses endothelin-1-induced vascular mitogenic triggering of atherosclerotic signaling (33). High concentrations of resveratrol inhibit angiotensin II-induced ERK1/2 phosphorylation and subsequent proliferation in cardiovascular smooth muscle cells (34). The role of resveratrol in inhibiting VEGF-induced endothelial cell proliferation, migration, invasion and angiogenesis (35). Resveratrol glycosides inhibit the formation of advanced glycosylation end products (AGEs), pentosidine (PTD) and prevent the accumulation of AGEs in the body, thereby treating AGEs-related diseases (36). Therefore, NO, ET-1,Ang II, VCAM-1, VEGF and PTD were used to assess the level of vascular endothelial function (37). The results also confirmed that *Dubosiella newyorkensis* has the same effect as resveratrol in improving vascular endothelial dysfunction. It was even superior to resveratrol in lowering VEGF and PTD in young mice, but there was no difference in aging mice, so *Dubosiella newyorkensis* has anti-primary aging potential. It has been shown that bacterial-derived metabolites can influence host longevity (38)and the gut microbiome releases an excess of small molecules. can bind genomic DNA directly and act as transcriptional co-regulators by recruiting transcription factors. thereby affecting the development of aging-related diseases (39). Resveratrol regulates trimethylamine oxide (TMAO) synthesis and bile acid (BA) metabolism by reshaping gut microbiota, thereby alleviating trimethylamine oxide-induced atherosclerosis (40). Resveratrol reshapes the gut microbiota of mice, including increasing the ratio of Bacillus-thick-walled bacteria, significantly inhibiting the growth of Prevotella, and increasing the relative abundance of *Bacillus*, *Lactobacillus*, *Bifidobacterium* and *Akkermansi* (41). Here we compared the effect of *Dubosiella newyorkensis* on gut microbiota and found that *Dubosiella newyorkensis* could increase beneficial genera such as *Lactobacillus, Bifidobacterium, Ileibacterium, Akkermansia, Staphylococcus, Verrucomicrobiota* and thus improve the gut microbiota. In summary, we believe that *Dubosiella newyorkensis* is an anti-aging drug with similar functions to resveratrol that improves oxidative stress, endothelial dysfunction and gut microbiota. *Dubosiella newyorkensis* could be an alternative or synergistic drug to resveratrol, offering new potential for future development of anti-aging drugs. The absence of differences in the first 20 predictions of gut microbiota function reinforces the similarity between *Dubosiella newyorkensis* and resveratrol.

Of course there are many shortcomings in this study, firstly, in terms of experimental design, the study did not establish a negative control. Only based on the initial study found that *Dubosiella newyorkensis* may have potential anti-aging effects, so the anti-aging wonder drug resveratrol was used as the standard to compare whether *Dubosiella newyorkensis* have similar functions in improving oxidative stress, vascular endothelial function, and regulation of gut microbiota, so as to investigate the positive effect of *Dubosiella newyorkensis* on aging. Secondly, in terms of selected indicators, only oxidative stress, vascular endothelial function, and gut microbiota affecting aging were selected as aging indicators, when in fact there are many indicators about aging. Finally, the experiment was only conducted in animal experiments, without clinical data to support.

## 5 Conclusion

In present study, by comparing the intervention of *Dubosiella newyorkensis* and resveratrol in young and aged mice, it was scientifically verified that *Dubosiella newyorkensis* and resveratrol have similar anti-aging functions. *Dubosiella newyorkensis* may even be superior to resveratrol in reducing oxidative stress, improving vascular endothelial function and improving gut microbiota distribution, which can be used as a novel anti-aging drug. It provides ideas and directions for clinical anti-aging and development of potential drugs.

## Data availability statement

The datasets presented in this study can be found in online repositories. The names of the repository/repositories and accession number(s) can be found below: https://www.ncbi.nlm.nih.gov/, PRJNA885520.

## Ethics statement

The animal study was reviewed and approved by the ethics committee of Jiangnan University approved the animal experiment.

## Author contributions

T-HL, C-YZ, G-ST, and Y-ZX participated in study design. T-HL, LZ, and Y-YS conducted animal experiment operation. JW and T-HL helped to draft and revise the manuscript. T-HL and JW carried out the statistical analysis of data. All authors contributed to the article and approved the submitted version.
